# Construction of immune-related LncRNAs classifier to predict prognosis and immunotherapy response in thymic epithelial tumors

**DOI:** 10.1042/BSR20220317

**Published:** 2022-05-13

**Authors:** Yongchao Su, Yangpeng Ou, Yongbing Chen, Ximiao Ma

**Affiliations:** 1 Department of Thoracic Surgery, Sanya Central Hospital, Sanya, 572000, Hainan Province, PR China; 2 Department of Oncology, Huizhou Third People’s Hospital, Guangzhou Medical University, Huizhou 516000, Guangdong Province, PR China; 3 Department of Thoracic Surgery, the Second Affiliated Hospital of Soochow University, Suzhou 215004, Jiangsu Province, PR China; 4 Department of Thoracic Surgery, Haikou People’s Hospital, Haikou 570208, Hainan Province, PR China

**Keywords:** immune infiltration, immunotherapy response, IRL classifier, prognosis, thymic epithelial tumors

## Abstract

The primary objective of this study was to construct an immune-related long noncoding RNAs (IRLs) classifier to precisely predict the prognosis and immunotherapy response of patients with thymic epithelial tumors (TET). Based on univariable Cox regression analysis and Lasso regression, six prognosis-related IRLs (AC004466.3, AC138207.2, AC148477.2, AL450270.1, HOXB-AS1 and SNHG8) were selected to build an IRL classifier. Importantly, results of qRT-PCR validated that higher expression levels of AC138207.2, AC148477.2, AL450270.1 and SNHG8 as well as lower expression levels of AC004466.3, and HOXB-AS1 in TETs samples compared with normal controls. The IRL classifier could effectively classify patients into the low-risk and high-risk groups based on the different survival parameters. In terms of predictive ability and clinical utility, the IRL classifier was superior to Masaoka staging system. Additionally, IRL classifier is significantly associated with immune cells infiltration (dendritic cells, activated CD4 memory T cells and tumor-infiltrating lymphocyte (TIL), T cell subsets in particular), immune microenvironment (immune score and immune checkpoint inhibitors) and immunogenicity (TMB) in TETs, which hints that IRL classifier is tightly correlated with immune characteristics and might guide more effective immunotherapy strategies for TETs patients. Encouragingly, according to TIDE algorithm, there were more immunotherapy responders in the low-risk IRL subgroup and the IRL score was robustly negatively linked to the immunotherapeutic response. To sum up, the IRL classifier was established, which can be used to predict the prognosis, immune infiltration status, immunotherapy response in TETs patients, and may facilitate personalized counseling for immunotherapy.

## Introduction

TETs (thymic epithelial tumors), which arise from the epithelial cells of the thymus, represent the most common neoplasms in the anterior mediastinum but are among the rarest of all cancers [1]. The most commonly used clinical staging systems and histological classification are Masaoka staging system and the World Health Organization (WHO), respectively, which exist to be a good correlation. According to the 2015 WHO classification, TETs are divided into thymoma (A, A/B, B1, B2, B3 subtypes) and thymic carcinoma (TC) (C subtypes) based on the tumor cell morphology, degree of atypia and extent of the thymocyte component. [2]. It has been reported that five-year median survival is about 66% in TC and reaches up to 90% in thymoma [3]. Surgical resection is considered the cornerstone curative treatment. However, local recurrence or distant metastasis may occur in some patients even after complete resection which render major obstacles to long-term survival in TETs [4]. Thus, identifying reliable biomarkers and establishing accurate predictive models is urgently necessary to optimize treatment regimens.

Immunotherapy strategies, such as the use of immune checkpoint inhibitors (ICI), have made remarkable progress and have gradually become promising first-line treatment choices in the field of oncology. To determine the full potential of this class of treatment options, clinical trials on the use of immunotherapy in different diseases, such as TETs, are ongoing [5]. However, since TETs are heterogenous, most patients do not respond well to immunotherapy due to primary or acquired resistance [6,7]. Unlike conventional therapy, the clinical benefits of immunotherapy are achieved by stimulating the sustained anti-tumor immune reaction [8], which relies on the immunoregulation between cancer cells and the tumor microenvironment (TME). Therefore, to help guide the selection and increase the efficiency of immunotherapy, more specific biomarkers and better predictive tools for screening the subset of patients with TETs who will respond to these strategies are needed.

LncRNAs are ubiquitous noncoding RNAs that are usually 200 nucleotides in length [9]. These molecules regulate 70% of human gene expression through their interactions with either DNA, RNA or proteins [10]. Furthermore, lncRNAs were also used in delivering therapeutic options and in estimating the prognosis of neoplasm patients [11]. Recently, an increasing number of evidence has revealed that lncRNAs can regulate the immune response by controlling the homeostasis, TME, anti-inflammatory factors and immune cell function [12,13]. For example, lncRNAs have been found to direct immune cell-specific gene expression, resulting in alterations in immune cell infiltration in cancer patients. In this study, we aimed to identify several new prognostic IRL markers and construct a classifier to precisely predict disease prognosis and immunotherapy efficiency in TETs.

The TCGA RNA-seq dataset and immune-related genes set were screened for IRL markers that were significantly correlated with prognosis outcomes. A classifier was then constructed using the identified IRLs. Subsequently, we estimated the predictive capacity and clinical utility of the IRL classifier and compared it against the Masaoka staging system. Then, we systematically correlated the IRL classifier with immunological characteristics and other relevant parameters, such as immune-related cells infiltration, TME scoring, microsatellite instability (MSI), tumor mutation burden (TMB), and immune checkpoint inhibitors. Finally, immunotherapy response and drug sensitivity of TET patients were predicted using the TIDE algorithm and CellMiner database respectively.

## Materials and methods

### Extraction of public data and data processing

We downloaded RNA-seq datasets, somatic mutation data, and corresponding clinicopathologic characteristics of TETs patients from a public TCGA database (https://gdc.cancer.gov/), which was recorded before September 10, 2021. A total of TETs 118 patients were collected with complete follow-up data, the clinical endpoint was disease-specific survival (DSS), recurrence-free survival (RFS) and overall survival (OS). The IRGs were acquired from gene set M19817 (immune response) and M13664 (immune system process) in MSigDB of Broad Institute (http://www.gsea-msigdb.org/gsea/index.jsp) [14]. The GSE29695 microarray dataset consisting of 37 TETs samples with clinical characteristics and gene expression profiles were collected from GEO (Gene Expression Omnibus) (https://www.ncbi.nlm.nih.gov/geo/). All data were normalized in the R computing environment using the edgeR package or Limma package. Data were utilized according to the data access policy of TCGA and GEO. All analyses were conducted in accordance with relevant regulations and guidelines.

### Clinical specimens

Twenty TETs tissues and paired adjacent normal tissues were collected from patients who underwent surgery. All tissue samples were blocked of formalin-fxed paraffin-embedded material and stored at 2–8°C with desiccation until use for later experiments. The study was reviewed and approved by the institutional review board (Ethics Committee) of Sanya Central Hospital.

### Identification of IRLs and construction of an IRL classifier

Mining and extraction methods of IRLs were described as previous studies [15]. Pearson correlation analysis using the cor test function of R (multiple corrected *P*-value < 0.05, correlation coefficient |Cor| > 0.4) was used in analyzing the expression levels of immune genes and lncRNAs in the datasets and obtaining a cohort of IRLs. The limma R package was used to identify differentially expressed IRGs (DEIRLs). The thresholds were set at a false discovery rate (FDR) value of <0.05 and a log fold-change (FC) value of >1. Univariable Cox regression analysis was used to screen prognosis-related IRLs (*P*<0.1). Using lasso regression analysis with 10-fold cross-validation and 1000 run cycles, candidate IRLs were identified. An IRL classifier was then constructed based on the called-out lncRNAs. Risk scores were calculated by a linear combination of coefficients from the lasso regression analysis and the relative expression of each IRL. Based on this formula, the IRL score for each clinical case was computed and was categorized into either low-risk or high-risk according to the median cut-off point. Survival differences (log-rank test) were compared by Kaplan–Meier survival analysis between low-risk and high-risk groups based on the different endpoints, including DSS, RFS and OS. Time-dependent ROC curves generated via the R package time ROC was used to assess the predictive performance of the classifier. Correlation analysis was performed between the risk scores and the different endpoints. Lastly, using the GSE29695 dataset, stratified analysis according to different clinical characteristics of patient samples (Masaoka staging I+II, Masaoka staging III+IV, TC and thymoma) was carried out to assess the discrimination capacity of the IRL classifier.

### Validation of IRL expression

Following the manufacturer’s protocol, Trizol (Invitrogen) was used to extract total RNA from formalin-fxed paraffin-embedded material. Reverse transcription of RNA using RevertAid RT Reverse Transcription Kit (Thermo Scientific). Quantitative PCR was performed using PowerUp™ SYBR™ Green Master Mix (Thermo Scientific). The results are standardized with GAPDH. Quantitative reverse transcription-PCR was conducted using the ABI 7500 real-time PCR system (Applied Biosystems, Foster City, CA, U.S.A.). Fold change was determined as 2-ΔΔCt in gene expression. Gene-specific PCR primers are listed in Supplementary Data S1.

### Determining the prognostic significance and clinical application of the IRL classifier

Univariate and multivariate Cox regression analyses were employed to investigate whether the predictive capacity of the IRL classifier is independent of other clinicopathological features of TETs patients in TCGA database, such as sex, age, sample initial weight, race, WHO histological type, tumor site, Masaoka staging, tumor status and mutation count. Additionally, ROC analysis using the R package survival ROC was used to compare the discrimination ability of the IRL classifier against the Masaoka staging system. Finally, decision curve analysis (DCA) via the stdca R package was carried out to estimate the net benefit and clinical usefulness of the IRL classifier in comparison to the Masaoka staging system [16].

### Evaluation of immune infiltration

The ESTIMATE algorithm was used to predict the presence of infiltrating immune or stromal cells in tumor tissues. This tool evaluates tumor purity using gene expression data based on single-sample gene set enrichment analysis (ssGSEA) and generated ESTIMATE score, immune score and stromal score. CIBERSORTx (https://cibersort.stanford.edu/) [17] was used to determine the relative abundance of the immune infiltrates. This R package is a kind of deconvolution algorithm which transforms the normalized gene expression matrix into the composition of infiltrating immune cells. Using this program, an accurate forecast of immune cell composition was done by filtering out samples with an output stringency set at *P*>0.05. Variance analysis of immune cells between high-risk and low-risk groups was visualized by constructing violin diagrams. The R package gene set variation analysis (GSVA) was employed to quantify the relative infiltration of immune cell subtypes based on the expression of immune-specific metagenes in ssGSEA. We focused on the metagene set of 16 immune cell types [18]. Two-tailed Wilcoxon test was used to analyze the immune scores and determine the differences in the predominant immune cell subtypes between the two groups (*P*<0.05). Vioplot R package was used to visualize the results.

### GSVA and ssGSEA

GSVA is a nonparametric unsupervised approach that converts genes from a sample matrix into pre-defined gene sets without a priori knowledge of experiment design. The Kyoto Encyclopedia of Genes and Genomes (KEGG) gene sets (c2.cp.kegg.v7.4.symbols.gmt), which was acquired from the Molecular Signatures Database (MSigDB) [19], were used to estimate the variation of pathway activity in each sample. The significantly enriched pathways in KEGG gene sets were set at a *P*<0.05 and an enrichment score change of > 1.0 between high-risk and low-risk groups. Additionally, ssGSEA was used to generate an enrichment score to signify the levels of absolute enrichment of a metagene set within a given dataset and to compare the differences in enrichment levels between high-risk and low-risk subgroups.

### Correlation analysis of tumor immunogenicity and immune checkpoint gene

TMB was computed by dividing the total covered bases in the genome with the total number of somatic mutations found. Meanwhile, MSI scores were collected from published studies [18]. Correlation analysis was conducted between the IRL classifier, TMB and MSI. We also explored the correlation between the IRL classifier and the expression levels via violin plot visualization.

### Immunotherapeutic response and drug sensitivity prediction using IRL classifier

The TIDE method (http://tide.dfci.harvard.edu) was considered to be a reliable algorithm for predicting the immunotherapeutic response of patients in recent studies [20]. We used this tool to evaluate the predictive efficiency of the IRL classifier in evaluating ICIs response in TETs. A TIDE score of <0 was recognized as positive sensitivity to immunotherapy, while a TIDE score of >0 was considered as negative sensitivity to immunotherapy. We then compared the rates of response between high-risk and low-risk groups. Furthermore, the IC50 data of over 20,000 compounds and the transcriptome for 60 cancer cell lines were downloaded from the CellMiner database v2.5 [21]. To ensure the clinical utility of IRLs, only those drugs with ongoing clinical trials and drugs with FDA approval were included in the analysis. Lastly, Spearman correlation analysis was conducted to determine the correlation between drug sensitivity and IRLs (|cor| > 0.3; *P*<0.01).

### Statistical analysis

SPSS statistics 22.0 and R software (R version 3.6.1) were used to performed the statistical analysis. A *P<*0.05 (two-sided) was considered statistically significant except for where a certain *P* value has been given.

## Results

### Construction and validation of the IRL classifier

**Figure 1:**
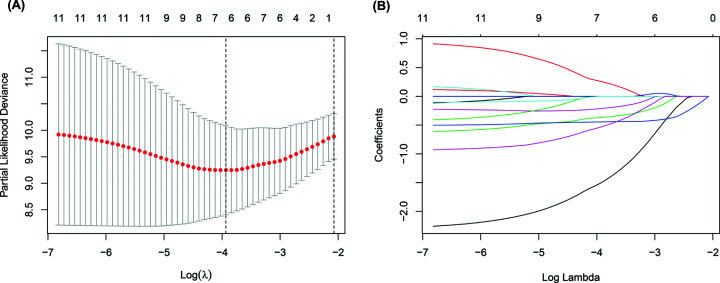
Six immune-related long non-coding RNAs (IRLs) selected by Lasso Cox regression analysis (**A**) The two dotted vertical lines are drawn at the optimal values by minimum criteria (left) and 1 - s.e. criteria (right). (**B**) Lasso coefficient profiles of the six IRLs. A vertical line is drawn at the optimal value by minimum criteria and results in six non-zero coefficients.

Using publicly available data repositories, 331 immune-related genes and 13,456 lncRNAs were selected from the MSigDB of Broad Institute and TETs cohort respectively. Co-expression networks between the selected immune-related genes and lncRNAs were assembled to identify IRLs. According to correlation coefficient |Cor| > 0.4 and multiple corrected *P*-value < 0.05, a total of 486 IRLs were identified in this study (Supplementary Material S2). Through differential analysis, we identified 84 DEIRLs between normal and tumor samples. From this, 12 prognosis-related IRLs were appraised using univariate Cox regression analysis (Supplementary Material S3). In constructing the IRL classifier, the lasso algorithm was used to shrink the obtained coefficients and select out six prognosis-related IRLs: AC004466.3, AC138207.2, AC148477.2, AL450270.1, HOXB-AS1 and SNHG8 (Figure 1). The computed IRL classifier risk score for each sample was used as a basis to group patients with TETs into a high-risk cohort and a low-risk cohort.

**Figure 2:**
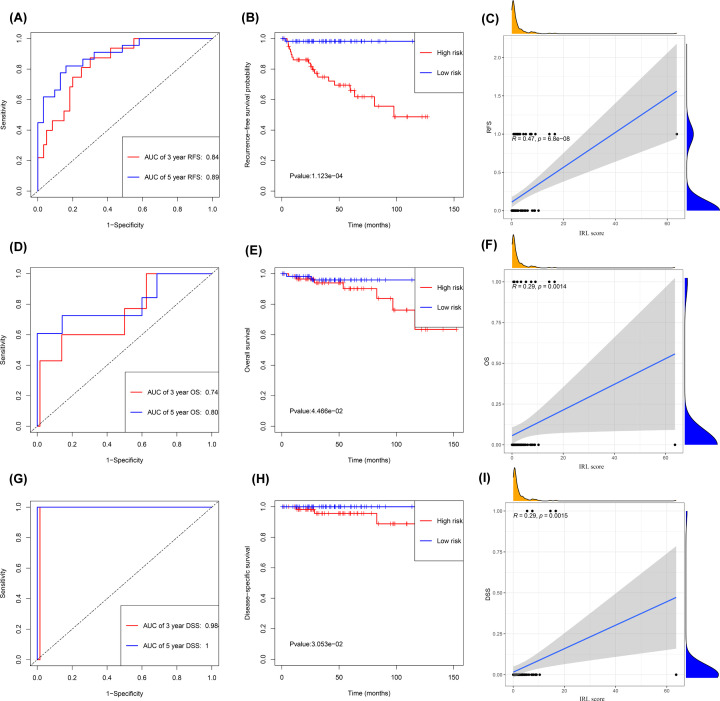
Development of IRL classifier for prediction of prognosis in TETs patients in TCGA database (**A**) Time-independent ROC curves with AUC values to evaluate predictive efficacy of IRL classifier based on RFS. (**B**) Kaplan–Meier estimates of patients’ RFS and time using the median risk score cut-off which divided patients into low-risk and high-risk groups. (**C**) Correlation between IRL score and RFS. (**D**) Time-independent ROC curves with AUC values to evaluate predictive efficacy of IRL classifier based on OS. (**E**) Kaplan–Meier estimates of patients’ OS and time using the median risk score cut-off which divided patients into low-risk and high-risk groups. (**F**) Correlation between IRL score and OS. (**G**) Time-independent ROC curves with AUC values to evaluate predictive efficacy of IRL classifier based on DSS. (**H**) Kaplan–Meier estimates of patients’ DSS and time using the median risk score cut-off which divided patients into low-risk and high-risk groups. (**I**) Correlation between IRL score and DSS.

**Figure 3:**
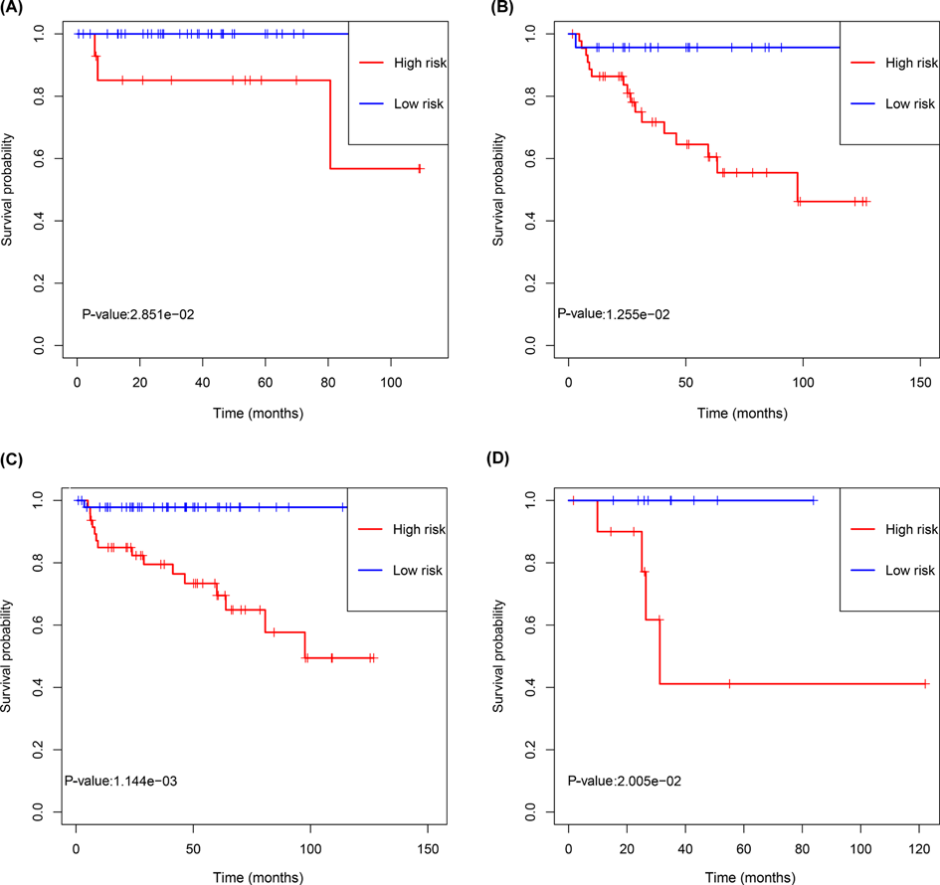
Development of IRL classifier for prediction of prognosis in TETs patients in TCGA database (**A**) Thymoma, (**B**) TC, (**C**) Masaoka staging I+II and (**D**) Masaoka staging III+IV. *P* values were calculated using the log-rank test.

**Figure 4:**
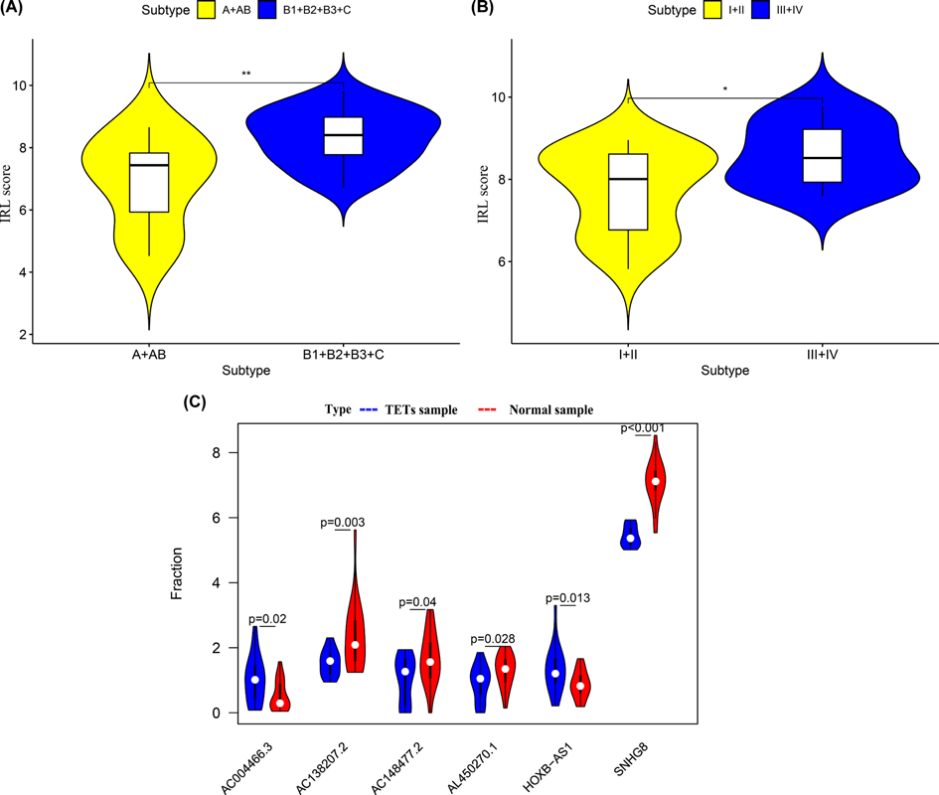
The predictive efficacy of IRL classifier in GSE29695 dataset and validation of IRL expression (**A**) Comparison of IRL score between A+AB subtypes and B1 + B2 + B3 + C subtypes. (**B**) Comparison of IRL score between Masaoka staging I+II and Masaoka staging III+IV. (**C**) Comparison of gene expression levels of 6 IRL genes between controls and TETs samples via qRT-PCR. Red indicates TETs sample, while blue indicates Normal sample.

Based on the median value of the IRL scores (Supplementary Figure S1B), a higher disease recurrence rate was observed in high-risk cohorts than in low-risk cohorts (Supplementary Figure S1A). Analyses of Kaplan–Meier curves showed that the RFS (*P*<0.001), OS (*P*=0.045) and DSS (*P*=0.030) of the patients in the low-risk group was significantly higher than that of patients in the high-risk group (Figure 2B,E,H). Time-dependent ROC curves showed that the IRL classifier had a high prediction ability, as evident from an AUC of 0.89 (5-year) and AUC of 0.84 (3-year) for RFS (Figure 2A); an AUC of 0.80 (5-year) and AUC of 0.74 (3-year) for OS (Figure 2D); and an AUC of 1.0 (5-year) and AUC of 0.98 (3-year) for DSS (Figure 2G). Consistent with these, correlation analyses results showed that the IRL score was significantly positively correlated with RFS, OS and DSS (Figure 2C,F,I). Furthermore, stratification analysis performed in subsets of patients according to different clinical factors (Masaoka staging I+II, Masaoka staging III+IV, TC and thymoma) using the IRL classifier revealed that the model is still able to effectively classify patients into high-risk groups and low-risk groups in the different subgroups (Figure 3). More importantly, in the GSE29695 dataset, the IRL scores were significantly lower in A+AB subtypes than in B1 + B2 + B3 + C subtypes (Figure 4A), and the IRL scores were also significantly lower in Masaoka staging I+II than in Masaoka staging III+IV (Figure 4B), which indicated that the IRL classifier was a robust risk model.

### Validation of IRL expression

To further validate the expression of the 6 IRL genes, we performed qRT-PCR in 20 clinical specimens. Compared with normal tissues, AC138207.2, AC148477.2, AL450270.1 and SNHG8 was significantly up-regulated in TETs samples (Figure 4C), yet the expression levels of AC004466.3, and HOXB-AS1 were significantly lower in TETs specimens. The detailed expression levels of 6 IRL genes was provided in Supplementary Data S4.

### Prognostic significance and clinical application of the IRL classifier

**Figure 5:**
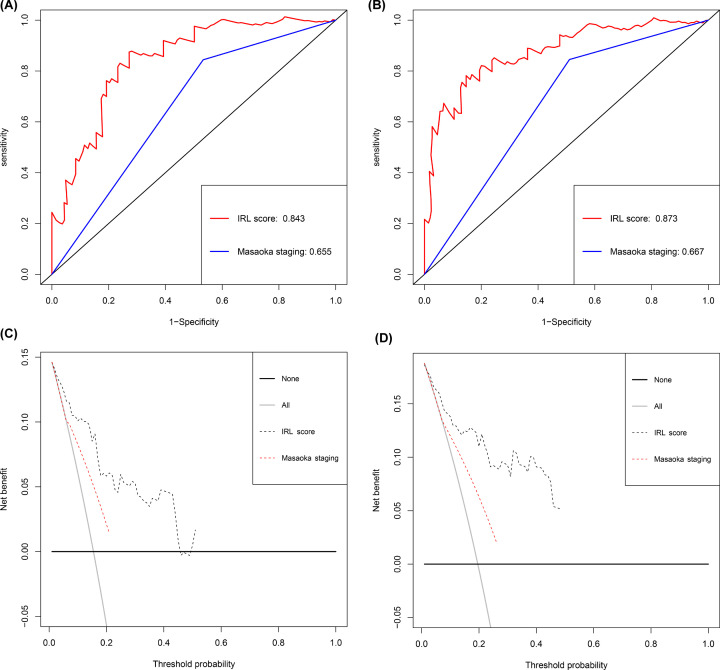
Prognostic significance and clinical application of the IRL classifier ROC curves compare the prognostic accuracy of the IRL classifier with Masaoka staging in predicting 3-year survival probability (**A**) and 5-year survival probability (**B**). Decision curve analysis for IRL classifier and Masaoka staging in the prediction of 3-year survival probability (**C**) and 5-year survival probability (**D**).

In determining whether the IRL classifier is independent of other clinical features, univariate Cox regression analysis found that WHO histological types, Masaoka staging, tumor status, and high IRL scores were related to shorter RFS (Table 1). Furthermore, multivariate Cox regression analysis confirmed that the IRL score was an independent predictor of poor prognosis, regardless of other clinicopathologic characteristics (HR: 1.06; 95%CI: 1.03–1.10; *P*<0.001). In evaluating the predictive capacity of the IRL classifier, ROC curve analyses showed that the AUC of the IRL classifier in predicting the 3-year and 5-year survival probability were 0.843 and 0.873, respectively, whereas that of the Masaoka staging system were 0.655 and 0.667 respectively (Figure 5A,B). Finally, the clinical value of the IRL classifier was estimated by DCA, which showed that the IRL classifier outperforms the Masaoka staging system at different cutoff times (3- and 5-year) (Figure 5C,D).

**Table 1:** Univariable and multivariable Cox regression analysis for prediction of RFS

Factors	Subgroup	Univariable analysis		Multivariable analysis	
		HR (95%CI)	*P*	HR (95%CI)	*P*
**Age**		0.99 (0.96–1.03)	0.692	NA	NA
**Sex**	Female	1			
	Male	0.60 (0.24–1.50)	0.276	NA	NA
**Race**	White	1			
	Black or african american	2.64 (0.60–11.67)	0.200	NA	NA
	Asian	0.33 (0.04–2.50)	0.281	NA	NA
**Sample initial weight**		1.00 (0.99–1.00)	0.320	NA	NA
**Tumor Site**	Thymus	1			
	Anterior mediastinum	1.73 (0.65–4.56)	0.271	NA	NA
**WHO histological types**	A-AB type	1			
	B-C type	3.72 (1.09–12.72)	0.037*	1.64 (0.42–6.34)	0.474
**Masaoka staging**	I-II	1			
	III-IV	1.42 (1.12–2.04)	0.030*	1.32 (0.81–2.08)	0.214
**Tumor status**	Tumor free	1			
	Tumor with	10.51 (4.12–28.86)	<0.001*	7.65 (2.76–21.21)	0.004*
**Mutation count**	<9	1			
	≥9	0.58 (0.22–1.52)	0.265	NA	NA
**IRL score**		1.06 (1.03–1.09)	<0.001*	1.06 (1.03–1.10)	<0.001*

Abbreviations: CI, confidence interval; HR, hazard ratio; RFS, recurrence-free survival.

NOTE: NA, not available. These variables were eliminated in the multivariate cox regression model, so the HR and *P* values were not available; *P<0.05.

### Estimation of immune cell infiltration

**Figure 6:**
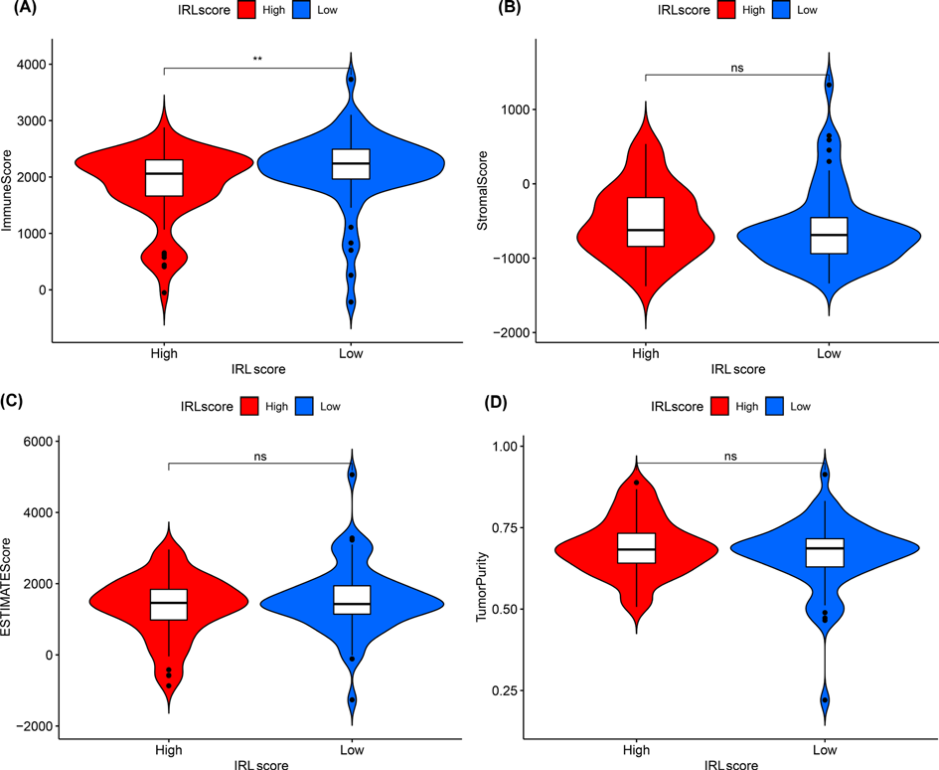
The comparison of the microenvironment score in high-risk group and low-risk group (**A**) Immune score, (**B**) stromal score, (**C**) ESTIMATE score and (**D**) tumor purity.

**Figure 7:**
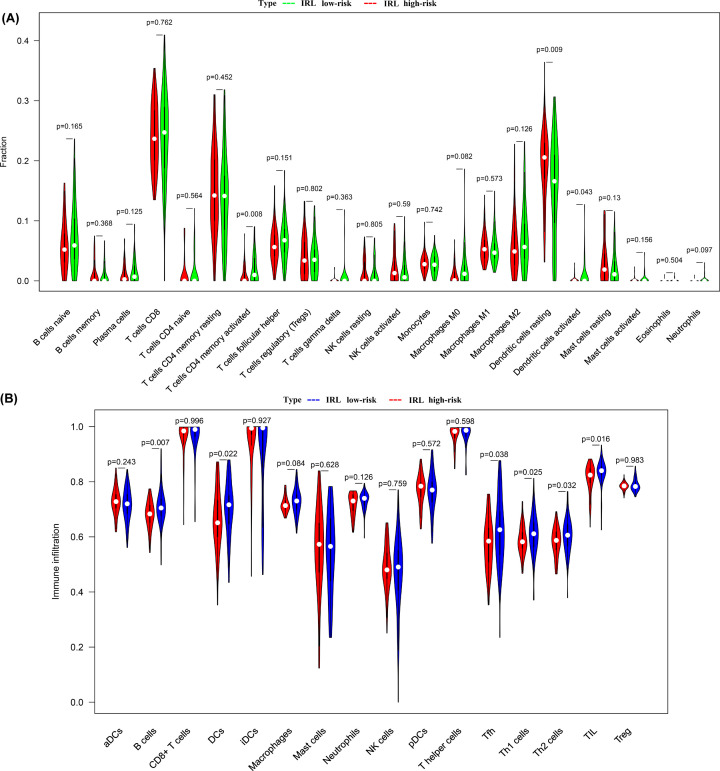
Comparison of infiltrating immune cells between high-risk group and low-risk group (**A**) CIBERSORTx tool. Red indicates high-risk, while green indicates low-risk. (**B**) ssGSEA. Red indicates high-risk, while blue indicates low-risk.

ESTIMATE analysis showed that the immune score of the high-risk group was lower compared with that of the low-risk group (*P*<0.001) (Figure 6A), while tumor purity, ESTIMATE score, and stromal score did not show any statistically significant difference (Figure 6B–D). We investigated the difference in infiltrating immune cells between the two groups. Using the CIBERSORTx program, our results demonstrated that activated CD4 memory T cells and dendritic cells were more abundant in the low-risk group while resting dendritic cells were more predominant in the high-risk group (Figure 7A). These findings reveal that immune-activated cells were significantly more abundant in the low-risk group. To complement our findings, we used ssGSEA, another cell-type quantification method, to quantify the enrichment scores of immune cell types. The ssGSEA results showed that B cells, dendritic cells, Tfh cells, Th1 cells, Th2 cells and TIL were significantly higher in low-risk groups (Figure 7B).

### GSVA and ssGSEA

**Figure 8:**
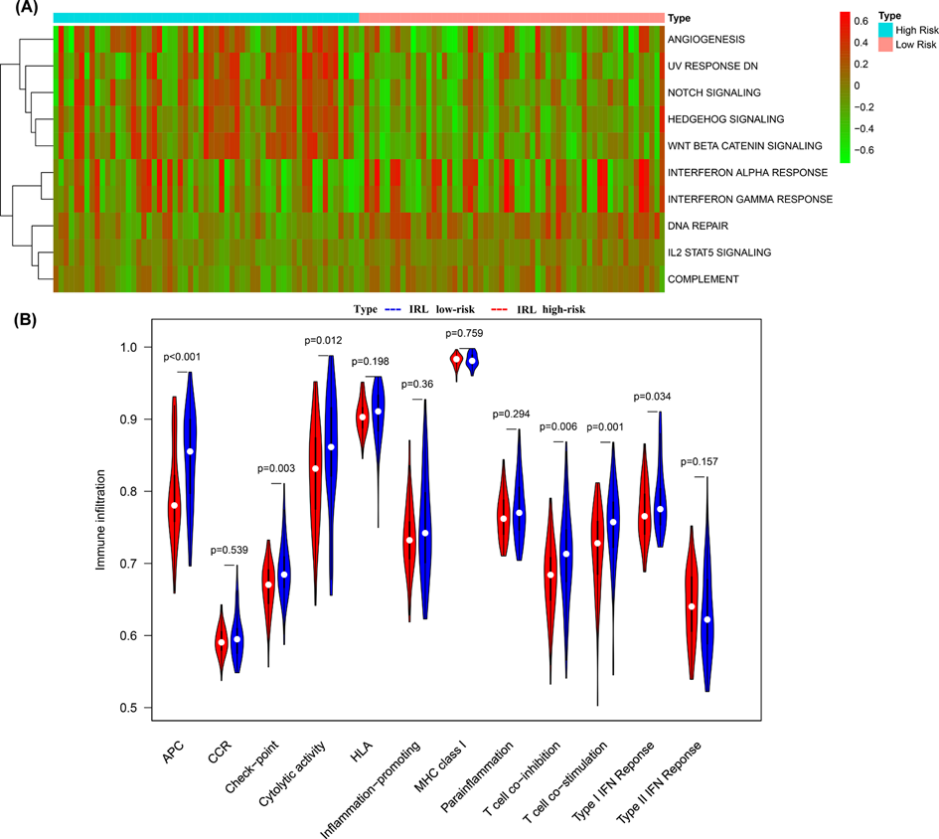
Potential biological pathways between high-risk and low-risk groups (**A**) Heatmap displays differences in pathway activities between high-risk group and low-risk group. (**B**) Comparison of immune-related pathway between high-risk group and low-risk group. Red indicates high-risk, while blue indicates low-risk.

To investigate the potential predominant biological pathways between high-risk and low-risk groups, we carried out GSVA based on KEGG gene sets. The heat map shows that Notch signaling, angiogenesis, hedgehog signaling, UV response DN and Wnt–β-catenin signaling were the main pathways involved in the high-risk group. Meanwhile, IL2 STAT5 signaling, interferon-gamma response, interferon-alpha response, complement and DNA repair were the main pathways associated with the low-risk group (Figure 8A). In addition, ssGSEA was utilized to explore the predefined immune-related pathways in the TETs. The ssGSEA results showed that APC, cytolytic activity, checkpoint, T cell co-stimulation, T cell co-inhibition and Type I IFN response were significantly associated with the low-risk group, indicating that the low-risk group was more involved in immune activation compared with the high-risk group (Figure 8B).

### Correlation analysis of tumor immunogenicity and immune checkpoint genes

**Figure 9:**
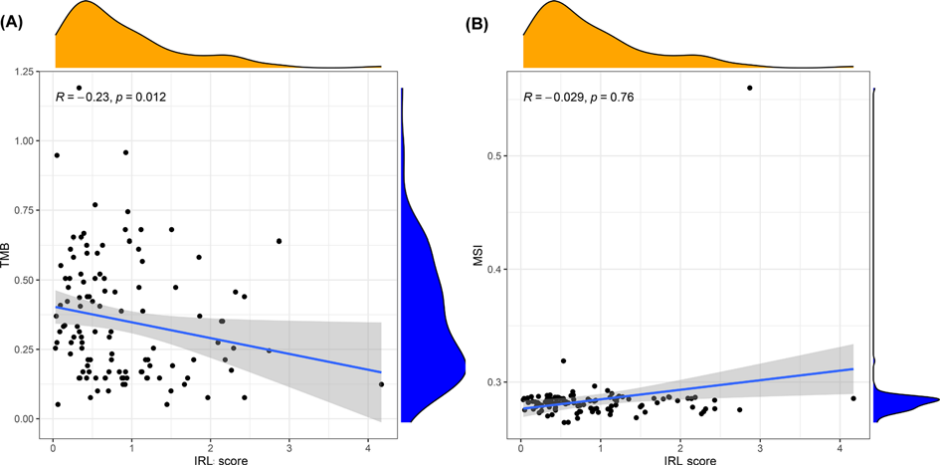
Correlation of IRL score and tumor immunogenicity (**A**) Correlation between IRL score and TMB. (**B**) Correlation between IRL score and MSI.

**Figure 10:**
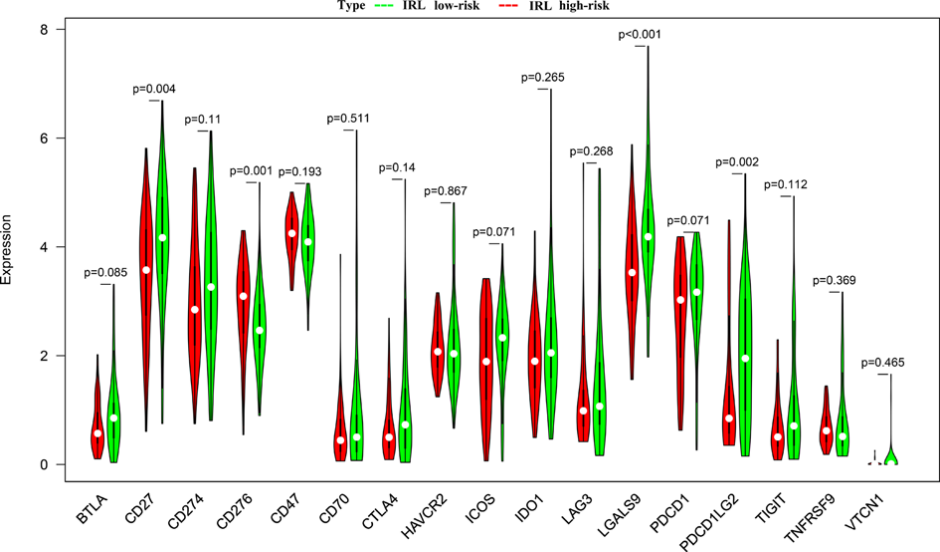
Analyzing immune checkpoint gene profiles Red indicates High-risk, while green indicates Low-risk.

We explored the relationship between tumor immunogenicity and IRL score. Our results showed that the IRL score was slightly negatively correlated with TMB (Figure 9A), while no significant correlation with MSI was observed (Figure 9B). To investigate the immune characteristics of patients with TET in both groups, we compared the expression levels of immune checkpoint genes between the low and high-risk cohorts. Our results showed that the low-risk group had significantly upregulated immune checkpoint gene profiles which include genes such as CD27, LAGLS9 and PDCD1LG2 (Figure 10).

### Immunotherapeutic response and drug sensitivity prediction using IRL classifier

**Figure 11:**
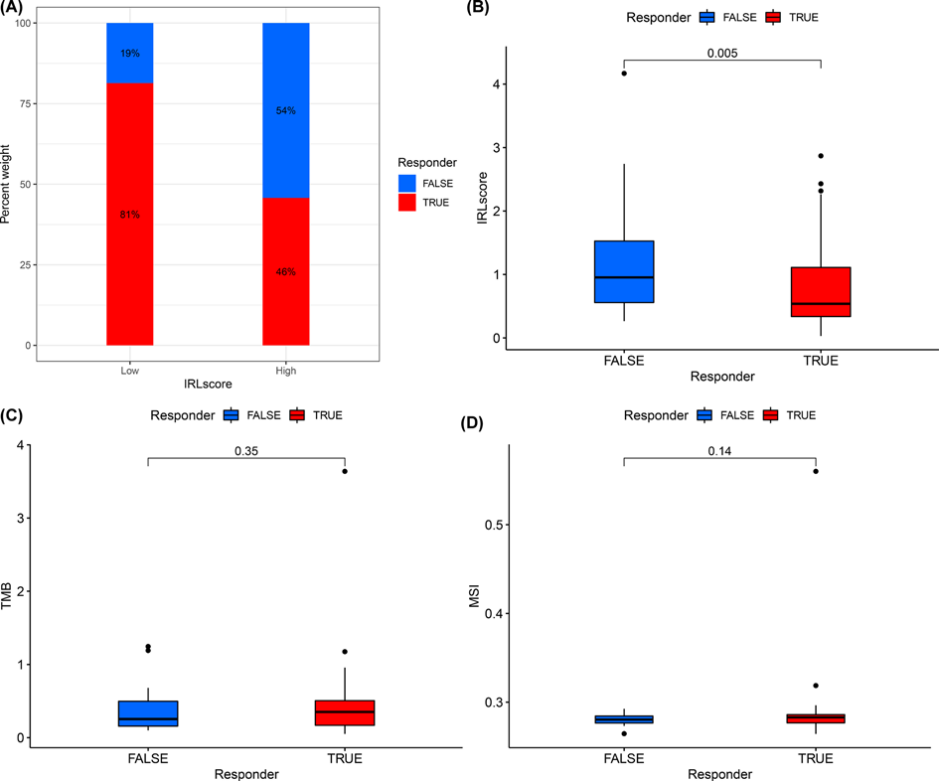
IRLs were efficient in prediction the immunotherapeutic benefit in TETs (**A**) The distribution of immunotherapeutic response in two groups stratified by IRL classifier in TETs cohort based on the TIDE algorithm. Two-sided Chi-square test was used to analyze contingency tables for ICIs responders. (**B**) Comparison of IRL score between responder group and nonresponder group. (**C**) Comparison of TMB between responder group and nonresponder group. (**D**) Comparison of MSI between responder group and nonresponder group.

**Figure 12:**
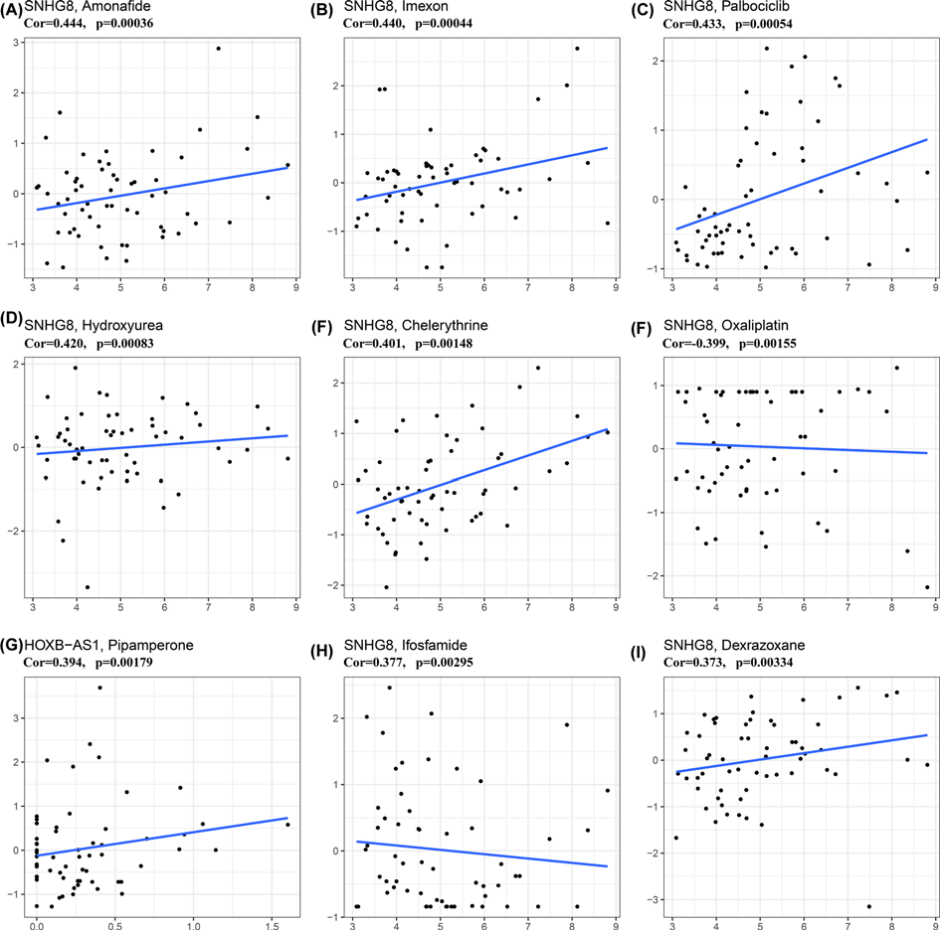
Correlation between IRLs (AC004466.3,AC138207.2, AC148477.2, AL450270.1, HOXB-AS1 and SNHG8) and drug sensitivity Correlation between IRLs (AC004466.3,AC138207.2, AC148477.2, AL450270.1, HOXB-AS1 and SNHG8) and drug sensitivity. (**A**) Amonafide. (**B**) Imexon. (**C**) Palbociclib. (**D**) Hydroxyurea. (**E**) Chelerythrine. (**F**) Oxaliplatin. (**G**) Pipamperone. (**H**) Ifosfamide. (**I**) Dexrazoxane.

TIDE was used to evaluate the potential clinical efficacy of immunotherapy in different IRL groups. The detailed output of the TIDE algorithm in the TETs cohort is shown in Supplemental Data S5. The number of immunotherapeutic responders was determined to be significantly higher in low-risk groups (48/59) compared with high-risk groups (27/59) (two-sided Chi-square test, *P*=0.001) (Figure 11A). In addition, the non-responders had higher IRL scores (*P*=0.005) (Figure 11B), while no statistically significant difference was observed for both TMB (*P*=0.35) and MSI (*P*=0.14) of the two response groups (Figure 11C,D). Finally, the correlation between IRLs (AC004466.3, AC138207.2, AC148477.2, AL450270.1, HOXB-AS1, and SNHG8) and antitumor drug sensitivity was explored using the CellMiner database. Forty-eight anticancer drugs that showed significant correlations with IRLs expression were screened (Supplementary Data S6). We found that SNHG8 expression was significantly positively correlated with the sensitivity to Amonafide, Imexon, Palbociclib, Hydroxyurea, Chelerythrine, Oxaliplatin, Ifosfamide and Dexrazoxane. Meanwhile, HOXB-AS1 expression was significantly negatively correlated with the sensitivity to Pipamperone, indicating a potential potent effect in treating TETs (Figure 12).

## Discussion

An increasing body of evidence suggests that IRLs can modulate the expression of immune-related genes involved in TME, immune cell differentiation and cancer immunity cycle [21–23]. In one study, it was found that NcRNA-RB1, a lncRNA expressed with an RB1 promoter, suppresses the expression of calreticulin, which is a calcium-binding chaperone protein. This in turn affects the presentation of the antigen to cytotoxic T cells and prevents tumor cells from releasing the ‘kill me’ signal [21]. Another study found that the interaction of small nucleolar RNA host gene 1 (LncRNA-SNHG1) with miR-448 negatively regulates the protein level of IDO to inhibit Treg cells differentiation in circulating peripheral blood and impede immune escape [22]. *In vivo* experiments exhibited that suppression of NEAT1, a nuclear paraspeckle localized lncRNA, by the miR-155/Tim-3 pathway reduces CD8^+^ T cell apoptosis and enhances active cytolytic function, thereby achieving immune activity [23]. In addition, Tim-3 was found to be up-regulated in chronic infection as well as in exhausted T cells in tumors. The increased Tim-3 expression results in CD8^+^ T cell death. Taken together, these studies indicate that IRLs participate in modulating the immune response and the cancer immunity cycle. Recent developments in immunotherapy strategies targeting ICIs have been applied in the clinical trial of TETs, yet inconsistent results have been reported [6,7]. Thus, it is necessary to investigate novel prospective prognostic IRLs markers, which may be useful for guiding the selection and improvement of immunotherapy.

To our knowledge, this is the first study conducted to identify new IRLs and establish an IRL classifier to precisely predict prognosis and immunotherapy response in TETs. Based on the co-expression networks and variance analysis, we were able to identify 84 DEIRLs. These IRLs were further subjected to multiple downstream analyses including univariable Cox regression analysis, modified lasso regression with multiple repeats and various cross-validation tests. Ultimately, we selected six IRLs (AC004466.3, AC138207.2, AC148477.2, AL450270.1, HOXB-AS1 and SNHG8) to develop a prognosis-related IRL classifier. More importantly, through gene expression analyses, we were able to validate that TET samples had higher expression levels of AC138207.2, AC148477.2, AL450270.1 and SNHG8 as well as lower expression levels of AC004466.3 and HOXB-AS1 compared with control samples. These findings are in line with the results of bioinformatics analyses using the TCGA datasets.

We report that the classifier can effectively group patients into either the low-risk group with longer survival rates or the high-risk group with shorter survival rates based on different survival parameters, including RFS, OS and DSS. Furthermore, stratification analysis performed in subsets of patients according to clinical factors (Masaoka staging I+II, Masaoka staging III+IV, TC and thymoma) showed that the IRL classifier can still effectively classify patients into high-risk groups and low-risk groups within different clinical subgroups. In GEO sets, the IRL classifier was able to distinguish between A+AB subtypes and B1 + B2 + B3 + C subtypes, as well as Masaoka staging I+II and Masaoka staging III+IV, further supporting the outstanding discrimination ability of the constructed IRL classifier.

Time-dependent ROC curves showed that the IRL classifier had a superior prediction ability. Consistently, correlation analysis results showed that the IRL scores were significantly positively linked to RFS, OS and DSS. Through univariate and multivariate Cox analysis we verified that the IRL classifier was an independent predictor of poor prognosis, regardless of other clinical features, indicating that the IRL classifier was a robust risk model. Currently, the Masaoka staging system is universally used to identify TET cases with high-risk for poor prognosis. In this study, we argue that the capability of the constructed model in predicting survival rates is superior to the Masaoka staging system. Interestingly, our DCA results demonstrated that survival-associated treatment decisions for TET patients based on the IRL classifier had a net beneficial effect compared with treatment decisions based on the Masaoka staging system, or treatment for all patients, or no treatment regime. To sum it all up, we suggest that the current IRL classifier may be helpful for clinicians in tailoring survival-related treatment decisions.

We identified six prognosis-related IRLs that were significantly different among the patients with TETs. HOXB-AS1and SNHG8 have been previously reported to be linked to cancers, such as glioblastoma, diffuse large B-cell lymphoma, multiple myeloma and colorectal cancer [24,25]. HOXB-AS1, a lncRNA situated in chromosome 17, played a tumor-promoting role in cancer. Bi et al. [24] revealed that HOXB-AS1 up-regulated the expression of HOXB2 or HOXB3 at the post-transcriptional and transcriptional levels through recruitment of ILF3 to driving the glioblastoma progression, which may be potential therapeutic molecules for patients with glioblastoma. SNHG8, is a member of the SNHGs (small nucleolar RNA host genes) family, was clarified as a critical driving force for the development of cancer. Research from Zhen et al. [25] uncovered that the expression of LncRNA SNHG8 was significantly increased in colorectal cancer tissue and cell line. Knockdown of SNHG8 via sponging of SNHG8 with miR-663 significantly promoted the migration, proliferation and invasion of colorectal cancer. Thus, further characterization of molecules such as AC004466.3, AC138207.2, AC148477.2, AL450270.1, HOXB-AS1 and SNHG8 in TETs would provide a novel perspective for the initiation and development of TETs, and assisted to find promising therapeutic targets for TETs patients.

The proportion and relative distribution of infiltrating immune cells in the TME are considered important factors in influencing cancer progression and immunotherapy response. Based on our ESTIMATE analysis, the immune score of the low-risk group was higher compared with the high-risk group, suggesting that the low-risk group is in a state of immune activation. In addition, the CIBERSORTx tool and ssGSEA algorithm were used for the first time to analyze the immune cell infiltration landscape in TETs. The CIBERSORTx findings showed that activated CD4 memory T cells and activated dendritic cells were more abundant in the low-risk group compared with the high-risk group. Analogously, the ssGSEA results revealed that compared to the high-risk group, dendritic cells, TIL, Tfh, Th1 cells and Th2 cells were significantly more predominant in the low-risk group. This finding suggests that immune-activated cells, such as dendritic cells [26] and T cells [27] were significantly more abundant in the low-risk group. These cells efficiently recognize antigens in killing tumor cells and enhance the effect of ICI immunotherapy.

Interestingly, GSVA results showed that signaling pathways related to tumor invasion and metastasis, such as angiogenesis, Notch signaling and Wnt β–catenin signaling, were the pathways mainly associated with the high-risk group. These pathways are recognized as immunosuppressive and have been reported to play a key role in tumorigenesis. On the other hand, immune response-related pathways, including interferon-gamma response and IL2 STAT5 signaling among others, were significantly activated in the low-risk group, which again underlines the significance of immune response modulation in TETs. Furthermore, the ssGSEA results showed that APC, Type I IFN response, checkpoint, T cell co-inhibition, cytolytic activity and T cell co-stimulation were significantly associated with the low-risk group, suggesting that the low-risk group is involved in immune activation and responds better to immunotherapy.

The immunogenicity of the tumor is the basis of initiating the antitumor immune response. In most cases, a higher frequency of somatic mutations leads to the production of more neoantigens by tumor cells, which in turn improves the immune killing ability of T cells via enhanced tumor cells recognition [28]. TMB, defined as the total number of somatic gene non-synonymous mutations, is considered an effective indicator in predicting tumor immunotherapy response [29]. In this study, we found that the IRL score was significantly negatively correlated with TMB, which hints that the IRL classifier may predict the efficiency of immunotherapy in TETs. Moreover, we observed that the expression of immune-checkpoints, such as CD27, LAGLS9 and PDCD1LG2, are significantly increased in low-risk patients, which might be attributed to the up-regulation of pre-existing TIL [30]. These findings prompted us to evaluate the potential of the IRL classifier in predicting ICIs response. Encouragingly, based on the TIDE analysis, there were more immunotherapy responders in the low-risk groups and the IRL classifier was robustly negatively linked to the immunotherapeutic response. Hence, the IRL classifier was proven to be efficient in predicting the immunotherapy response of patients with TETs. All of these findings indicate that the IRL classifier was a potent tool for determining the immunotherapy sensitivity of TET patients.

Given that TET cases with higher IRL scores may not be suitable for immunotherapy, it is necessary to explore several potentially effective drugs to target the prognosis-related IRLs (AC004466.3, AC138207.2, AC148477.2, AL450270.1, HOXB-AS1 and SNHG8). Using the CellMiner database, we identified several anticancer drugs for TETs, such as Amonafide and Pipamperone. However, the clinical safety and efficacy of those drugs against TETs remains to be explored. Therefore, it is urgent to confirm the therapeutic effect of these candidate drugs on TETs in further studies.

While significant, we recognize that our study has several limitations. First, we merely extracted and retrospectively analyzed imperfect data, such as TCGA datasets with relatively small sample sizes, and GEO datasets which not only lack follow-up data but also have a limited sample size, through biological algorithm approaches. This necessitates the external validation of our results using larger sample sizes and multi-center prospective cohorts. Second, while bioinformatics tools help exploit the discovery of novel biomarkers for diagnosis, treatment and prognosis, *in vitro* and *in vivo* experiments in TETs are also needed to further elucidate the molecular mechanisms of hub IRLs. Finally, patients with TETs did not receive corresponding ICIs treatment, and the immunotherapy response was evaluated using cutting-edge bioinformatics tools. However, although the IRL classifier can stratify TET patients with different immune responses, external data validation is lacking. Hence, multi-center, large-scale studies are needed to evaluate its usefulness in clinical trials and strengthen its clinical evidence.

## Conclusion

Taken together, we were able to construct an IRL classifier based on AC004466.3, AC138207.2, AC148477.2, AL450270.1, HOXB-AS1 and SNHG8 expression. We propose that this model can be used to predict the prognosis, immune infiltration status and immunotherapy response in TETs patients and may facilitate personalized counseling for immunotherapy.

## Supplementary Material

Supplementary Figure S1

Supplementary Data S1-S6

## Data Availability

The data that support the findings of this study are provided in supplementary materials and are also made available in the TCGA (https://gdc.cancer.gov/) and GEO (https://www.ncbi.nlm.nih.gov/geo/).

## Abbreviations

APC: antigen-presenting cell
AUC: area under curve
DCA: decision curve analysis
DC: dendritic cell
DEIRL: differential expression immune-related long noncoding RNA
DSS: disease-specific survival
FDR: false discovery rate
GEO: Gene Expression Omnibus
GSVA: gene set variation analysis
ICIs: immune checkpoint inhibitors
IRG: immune-related genes
IRL: immune-related long noncoding RNA
KEGG: Kyoto Encyclopedia of Genes and Genomes
LASSO: least absolute shrinkage and selection operator
lncRNA: long noncoding RNA
MSI: microsatellite instability
MSigDB: Molecular Signatures Database
OS: overall survival
RFS: recurrence-free survival
RNA-seq: RNA sequencing
ROC: receiver operating characteristic
ssGSEA: single-sample gene set enrichment analysis
TC: thymic carcinoma
TCGA: The Cancer Genome Atlas
TET: thymic epithelial tumor
TIDE: Tumor Immune Dysfunction and Exclusion
TIL: tumor-infiltrating lymphocyte
TMB: tumor mutation burden
TME: tumor microenvironment
WHO: World Health Organization
