# Supplementary material for: Construction of immune-related LncRNAs classifier to predict prognosis and immunotherapy response in thymic epithelial tumors
Source: Biosci Rep. 2022 May 13;42(5):BSR20220317. doi: 10.1042/BSR20220317 (PMC9109460; doi:10.1042/BSR20220317)
Supplement: Supplementary Figure S1 [file BSR-2022-0317_supp.pdf]

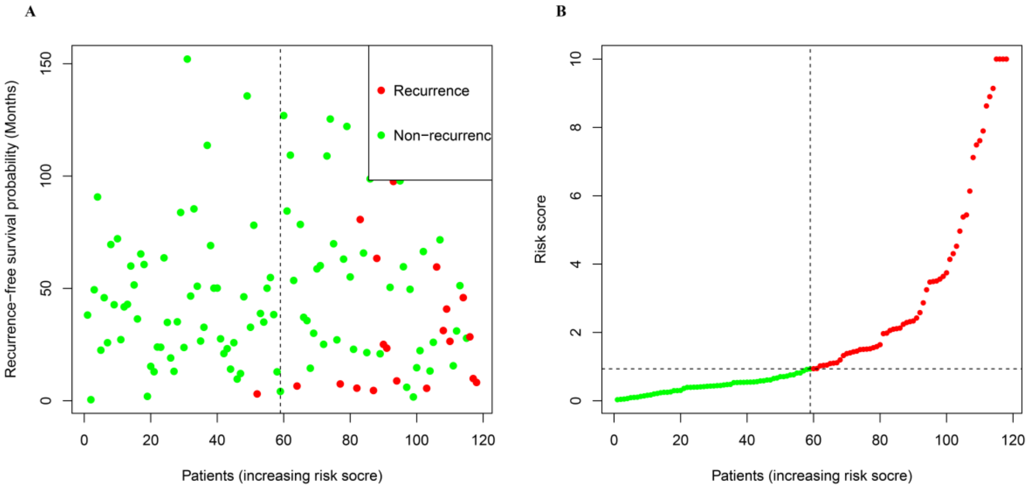

Figure S1. Development of IRL classifier for prediction of prognosis in TETs patients in TCGA database. (A and B) Distribution of IRL classifier risk score.
