# Supplementary material for: Construction of immune-related LncRNAs classifier to predict prognosis and immunotherapy response in thymic epithelial tumors
Source: Biosci Rep. 2022 May 13;42(5):BSR20220317. doi: 10.1042/BSR20220317 (PMC9109460; doi:10.1042/BSR20220317)
Supplement: Supplementary Data S1-S6 [file BSR-2022-0317_supp1.zip › BSR-2022-0317_supp1.pdf]

Supplemental Files

Supplementary material 1. Gene-specific PCR primers.

Supplementary material 2. Identification of IRLs in our research with ***p***-value < 0.05 and correlation coefficient |Cor| > 0.4.

Supplementary material 3. Using univariable Cox regression analysis, we identified 12 prognostic related IRLs.

Supplementary material 4. The detailed expression levels of 6 IRL genes.

Supplementary material 5. The detailed output of TIDE algorithm in TETs cohort

Supplementary material 6. Based on CellMiner database,48 anticancer drugs were significant correlation with IRLs expression.
